# As we wait: coping with an imperfect nomenclature for extracellular vesicles

**DOI:** 10.3402/jev.v2i0.20389

**Published:** 2013-02-15

**Authors:** Stephen J. Gould, Graça Raposo

**Affiliations:** 1 Department of Biological Chemistry, The Johns Hopkins University School of Medicine, Baltimore, MD, USA; 2 Institut Curie, Centre de Recherche, Paris, France; 3 Structure and Membrane Compartments CNRS, Paris, France

There is increasing evidence that secreted vesicles play important roles in numerous aspects of biology (e.g. intercellular vesicle traffic, immunity, development, neurobiology and microbiology), contribute to many human diseases (e.g. cancer, neurodegenerative disorders and HIV/AIDS) and have significant biotechnological potential. This expanding interest in extracellular vesicles has also highlighted some vexing problems related to their nomenclature. At the first meeting of the *International Society for Extracellular Vesicles* (ISEV) in Gothenburg, Sweden (April 2012), the authors chaired a session on the issue of vesicle nomenclature. Although it was not possible to reach a broad agreement on vesicle nomenclature, members of the session did reach consensus on 2 points. First, ISEV should strive to protect the scientific independence of its members on this issue. Second, that we (S.J.G. and G.R) should articulate some of the relevant points of concern in the *Journal of Extracellular Vesicles*.

## Nomenclature

Researchers have invented dozens of different names for secreted vesicles, most of which reflect specific functions (e.g. *calcifying matrix vesicles* that initiate bone formation (1) and *tolerosomes* that induce immunological tolerance to dietary antigens (2)) or their cell of origin (e.g. *dust* released by platelets (3) and *prostasomes* released by prostate epithelium (4)). Although such terms can be useful within a specialized field, more generic terms, such as “exosome” and “microvesicle”, have broader utility. Unfortunately, these generic terms mean different things to different investigators. For example, *exosome* can be used in 3 different ways, with some investigators preferring a biogenetic definition (i.e. vesicles that bud into endosomes and are released when the resulting multivesicular bodies fuse with the plasma membrane (5, 6)), others preferring the original, broad definition (i.e. secreted vesicles that “may serve a physiologic function” (7, 8)) and still others employing an empirical definition based on differential centrifugation (i.e. vesicles that sediment only after centrifugation at ~70,000–100,000×*g*) (5, 9). A similar range of definitions is evident for the term *microvesicle*, which some define as vesicles that bud from the plasma membrane (10, 11), others use to mean all secreted vesicles (12) and still others define on the basis of differential centrifugation (i.e. vesicles that sediment at ~10,000×*g*) (9, 11).

In the face of these conflicting definitions, we feel that investigators should not be forced to concede their scientific independence, violate precedent or ignore compelling empirical data when it comes to their choice of nomenclature. As such, we offer 4 suggestions for authors, reviewers and editors. First, authors should state their use of terms explicitly, choose their terms based on precedent and logical argument, and apply them consistently throughout a piece of work. Second, authors should clearly state their method(s) of vesicle collection, how the method(s) relate to their use of terms and even the method(s) for obtaining and storing biological fluids prior to isolating vesicles. Third, reviewers and editors should respect authors’ scientific freedom in their choice of vesicle nomenclature, so long as it follows precedent, logic and the authors’ data. Fourth, authors should be encouraged to use the term “extracellular vesicle” (EV) as a generic term for all secreted vesicles, and as a keyword in all publications.

## Vesicle size and morphology

The definitions for different classes of secreted vesicles often include physical properties, such as size and morphology. Early work established that cells secrete vesicles of 2 size classes, one of ~1 µm diameter and the other of ~100 nm (7). Later, the smaller vesicles were reported to have a narrow size range (~50–100 nm diameter), density and a cup-shaped morphology (5, 6). However, more recent studies in other cell types, and with other techniques, have shown that the small vesicles can have a broader size range and density (13, 14), and that the cup-shaped morphology was, in fact, an experimental artefact (15). We urge authors to take these considerations into account as they write their articles and grant proposals, and for reviewers and editors to do the same during the peer review process.

## Vesicle collection

It is human to name what we collect. As a result, it is natural for many to link the issue of vesicle nomenclature to the process of vesicle collection. The original, most scientifically sound, and widely used technique for separating and collecting secreted vesicles is differential centrifugation. In fact, many researchers use this technique to define the *microvesicle* (pellet at ~10,000×*g*) and *exosome* (pellet at~70,000–100,000×*g*) as separate classes of secreted vesicle. However, there is increasing concern within the field about the inappropriate application and interpretation of differential centrifugation in the analysis of extracellular vesicles, the variability introduced by the use of rotors with different *k* factors, the failure of investigators to note the *k* factor and the rotors used in their experiments, and the replacement of differential centrifugation by new and unverified techniques. To assuage these concerns, we urge investigators employing differential centrifugation techniques to include these minimal steps: a) removal of cells and large cell debris by low-speed centrifugation of the extracellular fluid (e.g. ~200–1,000×*g* for 3–15 minutes), b) pelleting of large, secreted vesicles from the cell-free supernatant by medium-speed centrifugation (e.g. 10,000×g for 30 minutes, a minimum of 2 times), c) collection of small, secreted vesicles by ultracentrifugation at~70,000–100,000×*g* and d) noting the *k* factor and type of rotor used in their experiments. While we do not argue that differential centrifugation is intrinsically superior to all alternative techniques for the collection and analysis of secreted vesicles, we do think that the long track record of this technique warrants its continued consideration as the “gold standard” against which all other techniques must be judged. Furthermore, we recommend the use of complementary biochemical experiments (e.g. sucrose density flotation gradient centrifugation, protease protection, light microscopy and electron microscopy) that can shed light on a protein's enrichment in extracellular vesicles, its topology in the vesicle and even the topology of the vesicles themselves.

## Perspective

There would be no need for this letter had the ISEV members agreed to a consensus nomenclature for extracellular vesicles. The current inability to reach consensus on several aspects of vesicle nomenclature reflects honest differences of opinion about the value of scientific precedent, the relative merits of empirical versus biogenetic systems for naming vesicles and scientific disagreement about the accuracy of current paradigms of vesicle biogenesis. We sincerely hope that the field will resolve these issues quickly, move towards a consensus nomenclature and limit the relevance of this letter to a brief window of time. To help in this regard, we urge the widespread adoption of the generic term EV when referring to secreted vesicles.
